# En Bloc Ovariohysterectomy for the Management of Feline Dystocia Due to Partial Primary Uterine Inertia: A Case Report

**DOI:** 10.1155/crve/7855852

**Published:** 2025-09-04

**Authors:** Ufaysa Gensa Geraro, Jiregna Dugassa Kitessa

**Affiliations:** ^1^Department of Animal Science, College of Agriculture and Natural Resource, Werabe University, Werabe, Ethiopia; ^2^Department of Veterinary Clinical Studies, College of Veterinary Medicine and Agriculture, Addis Ababa University, Bishoftu, Ethiopia

**Keywords:** emergency veterinary surgery, en bloc ovariohysterectomy, feline dystocia, uterine inertia

## Abstract

En bloc ovariohysterectomy is a surgical technique that involves the removal of the ovaries and uterus as a single unit, prior to the delivery of fetuses. A 4-year-old, nondescript cat named “Butulu,” weighing 2.5 kg, was presented to the Veterinary Clinic in Bishoftu, Ethiopia, with a history of dystocia during her second parity. The cat had delivered one dead kitten 19 h prior to presentation and was unable to deliver the remaining litter. Clinical signs included lethargy, depression, abdominal distension, and absence of straining. On external abdominal palpation, firm masses were detected bilaterally within the abdominal cavity. Based on the clinical history and physical examination, the condition was diagnosed as feline dystocia, attributed to partial primary uterine inertia. The patient underwent en bloc ovariohysterectomy under general anesthesia. The postoperative recovery was uneventful, and the cat exhibited complete clinical recovery 12 days after surgery.

## 1. Introduction

Dystocia is a reproductive emergency commonly encountered in small animals. The incidence of dystocia has been reported in approximately 5% of all parturitions in dogs and between 3.3% and 5.8% in cats [1, 2]. It may be caused by maternal factors, fetal factors, or a combination of both [3]. Among maternal causes, uterine inertia is the most common etiology of feline dystocia, accounting for nearly two-thirds of cases [4].

Uterine inertia refers to insufficient or absent uterine contractions during parturition. It may be associated with hormonal imbalances, electrolyte disturbances, or anatomical abnormalities such as pelvic deformities [5]. Complete primary uterine inertia is diagnosed when no signs of second-stage labor occur after the expected date of parturition. Partial primary uterine inertia is diagnosed when second-stage labor is initiated, but ineffective uterine contractions lead to the delivery of only one or a few fetuses, with failure to deliver the remainder [3, 6]. Other risk factors for dystocia in cats include breed predisposition, oversized fetuses, large litter size, and fetal malpresentation [7].

More than 60% of feline dystocia cases require cesarean section [8]. En bloc ovariohysterectomy is considered a surgical alternative to cesarean section in cases of dystocia, especially when future reproduction is not desired or if the dam's condition is critical [9]. This technique involves the removal of the entire uterus and ovaries with the fetuses still inside [10]. Robbins and Mullen [9] reported neonatal survival rates of 42% in cats and 75% in dogs following this technique, which is comparable to survival rates observed with standard medical and surgical management. However, the higher neonatal survival rate of 93.1% was seen following standard cesarean section [11].

En bloc ovariohysterectomy is contraindicated when fetuses are alive but exhibit signs of bradycardia or hypoxia, which may be due to compromised uterine blood flow [9, 10]. However, this technique is ideal in situations where the dam requires rapid surgical intervention, when the fetuses are nonviable, when the uterus is suspected to be infected, or when sterilization is desired [6]. When performing the procedure on a uterus containing live fetuses, the time between ligation of the blood supply and fetal delivery should not exceed 1 min to maximize neonatal survival [12, 13]. This case report presents the successful surgical management of dystocia due to partial primary uterine inertia in a 4-year-old cat using en bloc ovariohysterectomy.

## 2. Case Description

**Figure 1:**
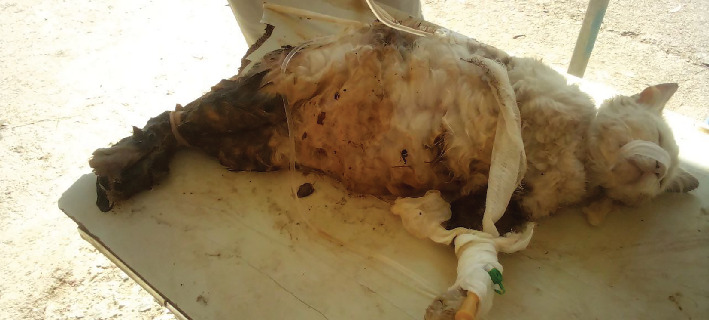
The cat received preoperative fluid therapy prior to surgery.

A 4-year-old, nondescript cat named “Butulu,” weighing 2.5 kg, was presented to the Veterinary Clinic of Addis Ababa University, College of Veterinary Medicine and Agriculture, Bishoftu, Ethiopia, with a history of dystocia during her second parity. According to the owner, the cat had delivered one dead kitten 19 h prior and had failed to deliver the remaining fetuses. Upon general physical examination, the cat appeared lethargic and depressed, lying in lateral recumbency with a soiled perineum. No active straining was observed. A small amount of reddish, malodorous vaginal discharge was noted, along with marked abdominal distension. On external abdominal palpation, firm masses were detected bilaterally. The cat was severely emaciated and dehydrated, as evidenced by a prolonged skin tent, and was administered preoperative fluid therapy (Figure 1). Vital signs included a body temperature of 38.1°C, respiratory rate of 22 breaths/min, and heart rate of 154 beats/min—within normal ranges, although the heart rate was elevated. Based on the clinical history and physical findings, a diagnosis of feline dystocia due to partial primary uterine inertia was made. Ultrasonographic examination and biochemical parameter assessment were not performed due to resource limitations.

### 2.1. Anesthesia Protocol

Preoperative medication included atropine sulfate administered intramuscularly at 0.04 mg/kg, followed by intravenous diazepam (Intas Pharmaceuticals Ltd., India) at 0.15 mg/kg for sedation. General anesthesia was induced using a combination of diazepam (0.15 mg/kg) and ketamine HCl (5 mg/kg, Germany) in the same syringe via IV injection. Anesthesia was maintained using intermittent bolus injections of half the induction dose at 5-to-10-min intervals, with anesthetic depth monitored manually throughout the procedure.

### 2.2. Surgical Procedure

**Figure 2:**
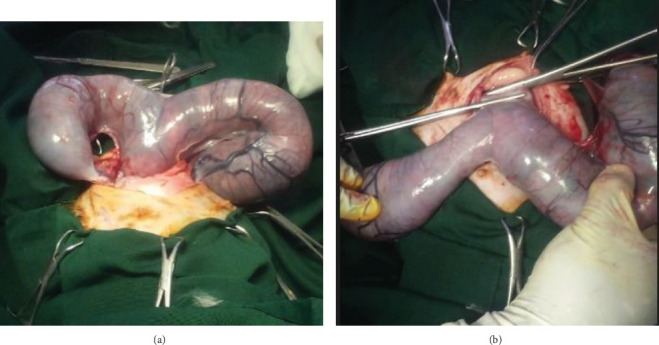
(a) Exteriorized uterus containing fetuses. (b) Two hemostatic clamps placed cranial to the ligature on the uterine body.

After placing in dorsal recumbency and gauze tying on the surgical table, a caudal ventral midline incision was made at the mid-third between the umbilicus and pubis, following the standard ovariohysterectomy approach [14]. The subcutaneous fat and connective tissues were bluntly dissected to expose the linea alba, which was incised to access the abdominal cavity. The gravid uterus was exteriorized (Figure 2a), and the abdominal viscera were packed with sterile gauze.

**Figure 3:**
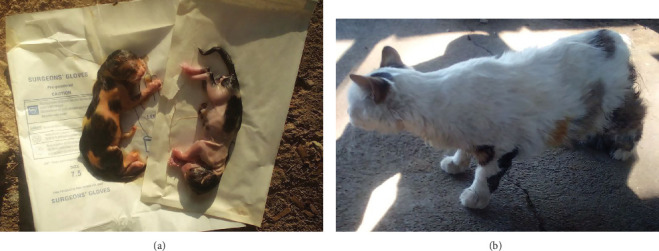
(a) Two dead male fetuses removed from the transected uterus. (b) The queen on 12th day postoperatively, showing signs of recovery.

The left uterine horn was traced cranially to the ovary. The suspensory ligament was transected sharply, and the ovarian pedicle was clamped, ligated using a double encircling ligature, and transected. Hemostasis was confirmed before returning the ligated stump to the abdomen. The right ovarian pedicle was similarly ligated and transected. Fenestrations were made in the broad ligament, and major vessels were ligated before transecting the ligament. The uterine body was exteriorized, and the cervix was palpated for retained fetuses. A circumferential ligature was placed at the uterocervical junction, and the uterine vessels were ligated separately. Two clamps were placed above the ligature (Figure 2b), and the uterus was transected between them. The assistant immediately incised the uterus and removed two dead male fetuses (Figure 3a). The uterine stump was checked for hemorrhage and returned to the abdominal cavity. The abdominal wall, including peritoneum and linea alba, was closed using 2-0 polyglycolic acid (Vicryl) in a continuous Ford interlocking suture pattern. Subcutaneous tissues and skin were closed in separate layers. The total duration of the surgery was approximately 30 min.

### 2.3. Postoperative Management

Postoperatively, a combination of procaine penicillin (24 mg/kg) and dihydrostreptomycin sulfate (30 mg/kg) (Pen & Strep, Norbrook, UK) was administered intramuscularly for five consecutive days. Analgesia was provided using tramadol hydrochloride at a dose of 2 mg/kg once daily for 3 days. Twenty-four hours after surgery, the cat exhibited signs of lethargy and anorexia. Supportive therapy was initiated with 2.5% dextrose glucose and lactated Ringer's solution for 2 days, until voluntary food and water intake resumed. The surgical wound was managed with daily application of diluted 5% tincture of iodine for 5 days. Additionally, a locally available plastic washbasin was used as an improvised Elizabethan collar to prevent licking or biting at the surgical site. The owner was advised to monitor the cat closely for any signs of postoperative complications or discomfort. By postoperative Day 12, the cat had recovered fully without any notable complications (Figure 3).

## 3. Discussion

Unlike large domestic mammals, the incidence of dystocia in cats is relatively low, accounting for approximately 3.3% to 5.8% of all parturitions [1, 2]. Although several studies have addressed the incidence and management of dystocia in cats, the majority of cases (67.1%) are attributed to maternal causes, with fetal factors accounting for 29.7% of cases [3]. Among maternal causes, uterine inertia is the most common, contributing to nearly two-thirds of feline dystocia cases [4]. Partial primary uterine inertia specifically accounts for approximately 40% of all uterine inertia cases [9]. In the present case, dystocia was diagnosed as being due to partial primary uterine inertia.

Partial primary uterine inertia occurs when the cat is able to deliver the first fetus through the pelvic canal, indicating some initial uterine contractility. However, exhaustion of the uterine musculature results in a loss of contractile strength, and the queen becomes unable to deliver the remaining fetuses [3, 4]. Literature suggests that dystocia is more commonly observed in purebred cats, particularly Persians and Birmans [4, 15]. However, in this report, the affected cat was a nondescriptive domestic cat.

The principle of en bloc ovariohysterectomy involves the removal of the ovaries and uterus as a single unit, before the fetuses are extracted [9, 12]. In the current case, this technique was applied by first clamping and ligating the ovarian and uterine arteries, followed by the removal of the entire uterus, which was handed off to the assistant team. Two dead male kittens were subsequently removed. En bloc ovariohysterectomy is considered the preferred surgical option in cases where the uterus is suspected to be infected and the fetuses are nonviable, as it may offer the best chance of saving the dam's life [6]. This finding is supported by the clinical presentation in the current case, where the cat exhibited signs of delayed second-stage labor, a history of expelling a dead fetus 19 h prior, and foul-smelling vaginal discharge. Foul-smelling discharge in feline dystocia is indicative of prolonged parturition, often due to lack of timely veterinary intervention (Iris and [16]) or the development of maternal toxemia [5].

Although Robbins and Mullen [9] reported an increased risk of maternal morbidity associated with ovariohysterectomy performed during dystocia, no severe postoperative complications were observed in the present case apart from transient anorexia and lethargy. The en bloc ovariohysterectomy offers advantages in reducing anesthesia duration, particularly in debilitated patients, and minimizes the risk of peritoneal contamination with uterine contents [6]. Additionally, it eliminates the need for a future sterilization procedure, which is beneficial for owners who may not be able to afford a second surgery. This technique, however, is contraindicated in cases where fetuses are alive but show signs of bradycardia or hypoxia, as these conditions may further compromise neonatal survival [10]. Further analytical research is required to evaluate the efficacy of en bloc ovariohysterectomy in cases involving both stressed and nonstressed fetuses.

## 4. Conclusion

En bloc ovariohysterectomy appears to be a safe and effective technique for the management of dystocia due to partial primary uterine inertia, particularly under field conditions in Ethiopia. It offers several advantages, including reduced surgical time, minimized risk of infection, and elimination of the need for a second sterilization procedure.

## Data Availability

All the data are presented in the document.
